# The accuracy of initial diagnoses in coma: an observational study in 835 patients with non-traumatic disorder of consciousness

**DOI:** 10.1186/s13049-020-00822-w

**Published:** 2021-01-12

**Authors:** Maximilian Lutz, Martin Möckel, Tobias Lindner, Christoph J. Ploner, Mischa Braun, Wolf Ulrich Schmidt

**Affiliations:** 1 Department of Neurology, Charité – Universitätsmedizin Berlin, corporate member of Freie Universität Berlin, Humboldt-Universität zu Berlin and Berlin Institute of Health, Augustenburger Platz 1, 13353 Berlin, Germany; 2 Department of Emergency Medicine, Charité – Universitätsmedizin Berlin, corporate member of Freie Universität Berlin, Humboldt-Universität zu Berlin and Berlin Institute of Health, Berlin, Germany; 3 Center for Stroke Research, Charité – Universitätsmedizin Berlin, corporate member of Freie Universität Berlin, Humboldt-Universität zu Berlin and Berlin Institute of Health, Berlin, Germany

**Keywords:** Coma, Diagnostic errors, Diagnostic accuracy, Emergency medical services, Disorder of consciousness

## Abstract

**Background:**

Management of patients with coma of unknown etiology (CUE) is a major challenge in most emergency departments (EDs). CUE is associated with a high mortality and a wide variety of pathologies that require differential therapies. A suspected diagnosis issued by pre-hospital emergency care providers often drives the first approach to these patients. We aim to determine the accuracy and value of the initial diagnostic hypothesis in patients with CUE.

**Methods:**

Consecutive ED patients presenting with CUE were prospectively enrolled. We obtained the suspected diagnoses or working hypotheses from standardized reports given by prehospital emergency care providers, both paramedics and emergency physicians. Suspected and final diagnoses were classified into I) acute primary brain lesions, II) primary brain pathologies without acute lesions and III) pathologies that affected the brain secondarily. We compared suspected and final diagnosis with percent agreement and Cohen’s Kappa including sub-group analyses for paramedics and physicians. Furthermore, we tested the value of suspected and final diagnoses as predictors for mortality with binary logistic regression models.

**Results:**

Overall, suspected and final diagnoses matched in 62% of 835 enrolled patients. Cohen’s Kappa showed a value of κ = .415 (95% CI .361–.469, *p* < .005). There was no relevant difference in diagnostic accuracy between paramedics and physicians. Suspected diagnoses did not significantly interact with in-hospital mortality (e.g., suspected class I: OR .982, 95% CI .518–1.836) while final diagnoses interacted strongly (e.g., final class I: OR 5.425, 95% CI 3.409–8.633).

**Conclusion:**

In cases of CUE, the suspected diagnosis is unreliable, regardless of different pre-hospital care providers’ qualifications. It is not an appropriate decision-making tool as it neither sufficiently predicts the final diagnosis nor detects the especially critical comatose patient. To avoid the risk of mistriage and unnecessarily delayed therapy, we advocate for a standardized diagnostic work-up for all CUE patients that should be triggered by the emergency symptom alone and not by any suspected diagnosis.

## Background

Emergency assessment of comatose patients, i.e., patients who do not present with an obvious cause such as cardiac arrest or traumatic brain injury, is a challenge both in the emergency department (ED) and in the prehospital setting. Up to 2% of all emergency patients present with coma of unknown etiology (CUE) [1, 2]. CUE differs from other emergency symptoms, as there is a vast number and diversity of underlying disorders, ranging from internal medicine to neurology and neurosurgery. Management of many of these disorders is highly time critical. Moreover, unconsciousness has proven to be a predictor for mortality and severity of the underlying pathology [3–6]. Thus, CUE requires an early and extensive diagnostic work-up leading to a reliable diagnosis and targeted treatment. During work-up, rapid differentiation between primary damage and secondary affection of the central nervous system (CNS) is a vital step since they require different hospital resources [7].

The assessment of the comatose patient begins in the prehospital phase and continues in the emergency department [8]. During first evaluation, emergency care providers frequently formulate – explicitly or implicitly – an initial diagnosis which often determines the first approach to the patient in the ED in order to benefit from a fast treatment [9, 10]. In CUE, the initial diagnosis often relies on sparse information, which may lead to errors and misdirect emergency management.

Two questions arise: First, are initial diagnoses in CUE sufficiently reliable? And second, if not, are initial diagnoses in CUE associated with any systematic flaws? The epidemiological and etiological description of patients with CUE in emergency settings is surprisingly scant [10, 11]. One study describes the epidemiology and initial diagnoses of prehospital patients with impaired consciousness, albeit without a systematic comparison of the initial diagnosis and the final diagnosis [12]. Another study examines the accuracy of the initial diagnosis in the more general population of patients in altered mental status [13]. To our knowledge, there is as yet no systematic study on the accuracy of initial diagnoses in patients with CUE.

## Methods

In the present retrospective observational study, we report on the accuracy of initial diagnoses made by prehospital emergency care providers in emergency patients presenting with CUE. Applying a standardized routine, we compared the pre-hospitally recorded initial diagnoses with the finally diagnosed CUE-explaining pathologies.

### Setting

Charité Campus Virchow-Klinikum is a tertiary care university hospital located in the north-western city center of Berlin, Germany, caring for approximately 70.000 emergency patients per year. Around 4100 patients per year present with a neurological chief complaint [14], numbers continuously increasing. A public emergency medical services (EMS) agency (“Berliner Feuerwehr”) coordinates emergency prehospital care in Berlin. It deploys paramedics that may be accompanied by an emergency physician according to the suspected severity of the called-in emergencies. In Germany, paramedics (“Notfallsanitäter”) have completed a three-year training. Legally, they are not allowed to state a diagnosis, however they may formulate a working hypothesis or preliminary, suspected diagnosis to notify the emergency department. Furthermore, drug administration is legally restricted to physicians, however in cases of emergency, paramedics may perform some defined procedures (general delegation). The local EMS agency defines these standard procedures. Emergency physicians’ education in Germany consists of 6 years in medical school, then at least two years of medical practice in hospital plus a dedicated emergency physician training and board certification.

### Ethics

An ethics vote for the analysis of routinely acquired clinical data was obtained from the Ethics Committee of the Charité-Universitätsmedizin Berlin (EA2/100/18: “Neurological leading symptoms in the Emergency Department”).

### Patient enrolment

**Fig. 1:**
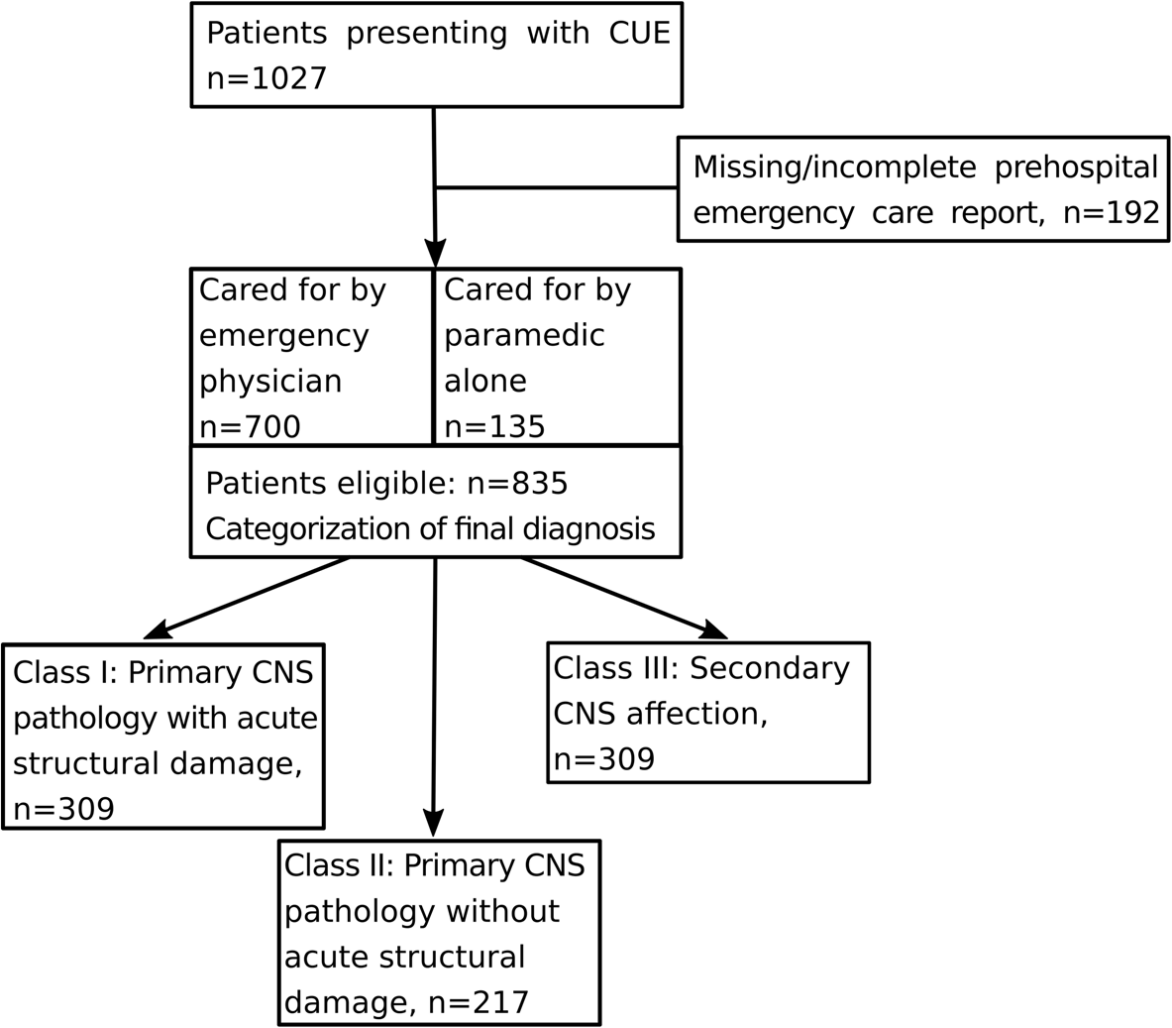
Patient Enrolment Flow and distribution in final diagnoses (grouped and classified)

Between May 2013 and January 2017, we prospectively identified all emergency patients presenting with CUE to the ED (*n* = 1027). Comatose patients with severe head trauma/traumatic brain injury (TBI) or primary cardiac arrest were not enrolled. By definition, these pathologies were considered obvious and unmistakable causes of coma and patients were treated along different and specific pathways. We determined unconsciousness by the inability to communicate verbally or by eye, head or limb movements, accompanied by Glasgow Coma Score (GCS) assessment [7]. Fully documented prehospital emergency care reports were available in 835 of 1027 patients from which we could obtain initial diagnoses/ hypotheses. Figure 1 shows the composition of the study cohort and the distribution of final diagnoses [15].

### Data preparation

Investigated variables were GCS at first contact with the comatose patient, type of emergency care provider (emergency physician or paramedic), categorized initial diagnosis (or working hypothesis, respectively), final diagnosis, in-hospital mortality, age and sex. GCS values were binned into three groups: GCS 3–5: low, 6–8: moderate and 9–12: high. We obtained GCS values and diagnoses/hypotheses from two sections in the standardized report forms used by prehospital emergency care providers. In the first section, emergency care providers put down a short summary (prose) in which they usually name the reasons for their diagnoses/hypotheses. Here, abbreviations were not always used clearly by emergency care providers, so we applied the common denominator (e.g., “ICH” was interpreted as intra*cranial* haemorrhage rather than intra*cerebral* haemorrhage). The second section provides a list of frequent and explicitly named diagnoses that may be ticked. Using the information from both sections, we established 13 groups of initial diagnoses/hypotheses (e.g., cerebral haemorrhage or ischemia, intoxication, pulmonary disease) to compare these with the final diagnoses. We expected to have more initial diagnoses/hypotheses than final diagnoses, since prehospital care providers may have stated more than one suspected pathology or several differential diagnoses.

Furthermore, these groups of diagnoses/hypotheses were categorized by their patho-physiology into three main classes: I) primary CNS pathology with acute structural brain damage, II) primary CNS pathology without acute structural brain damage, III) acute secondary CNS affection. Comatose patients without any specified initial diagnosis/hypothesis (explicitly stated as unspecified by the emergency care provider) were labelled class IV. While Plum and Posner had categorized causes of coma into only two categories (primary or focal vs. secondary or diffuse) [7], we used a modified classification by splitting primarily CNS-related coma-explaining pathologies into primary causes with acute structural CNS damage (e.g. intracranial haemorrhage) and primary causes without acute CNS damage (e.g. epilepsy). A primary CNS pathology with acute structural brain damage (class I) is diagnosed by imaging and/or CSF-testing and may require an urgent invasive intervention. A detailed neurological assessment provides the basis for identifying class II diagnoses that usually require less specialized hospital resources. Secondary pathologies (class III) often require a combination of many diagnostic tools (history, examination, ECG, laboratory, imaging) and a variety of mostly conservative treatment options.

By definition, patients were grouped into class I whenever prehospital care providers suspected an acute structural brain damage since classes of diagnoses differ in mortality [16] and final diagnoses from class I need to be excluded urgently.

Patients with epileptic seizures/epileptic states or post-ictal loss of consciousness were grouped into class II when there was no suspicion of acute structural brain damage or other triggers for acute-symptomatic seizures. Accordingly, class II included other primary CNS pathologies without suspicion of acute structural brain damage such as neuro-degenerative diseases or psychiatric disorders (e.g., “pseudo-coma” in dissociative or akinetic states). Class III was reserved for medical disorders, intoxication and rare surgical emergencies that affected the CNS secondarily [15].

Emergency care providers might have recorded more than one initial diagnosis/hypothesis without stating a causality between them. For example, an epileptic seizure could have been an acute-symptomatic seizure or the symptom of epilepsy. Whenever a pathology from class I was mentioned among multiple initial diagnoses/hypotheses, patients were grouped into class I. The remaining patients with more than one initial diagnosis/hypothesis were grouped into class III. Pathologies that do not explain coma were not considered (e.g., minor bone fractures).

### Final diagnoses

In order to ascertain the final diagnoses underlying CUE in the study cohort, two board-certified physicians (MB, WS) had reviewed each patient’s clinical, laboratory, radiology and autopsy findings (if available) after discharge, death or transfer to a rehabilitation facility. One consensual main diagnosis had been allocated to each patient. Final diagnoses were classified similarly: I) primary CNS pathology with acute structural brain damage, II) primary CNS pathology without acute structural brain damage, III) acute secondary CNS affection [15].

### Data analysis

Descriptive statistics and demographics are reported as medians and interquartile ranges. The match/mismatch between initial diagnoses/working hypotheses and final diagnoses is presented as ‘percent agreement’. To account for guessing and artificial inflation of percent agreement, we further tested with Cohen’s Kappa [17]. To be accurate using Cohen’s Kappa, we had to omit class IV (CUE without initial diagnosis/hypothesis). Differences in the spectrum of initial diagnoses between groups with different Glasgow coma scores were tested by χ^2^-test and Cramér V correlation coefficient (range, 0 [no correlation] to 1.00 [strong correlation]) [18]. Binary logistic regression was performed to investigate classes of initial diagnoses/working hypotheses, classes of final diagnoses, GCS-bins, age and sex as predictors for in-hospital mortality. Odds Ratios (OR) for each significant variable are reported with their 95% confidence interval (CI). A *p*-value below .05 was considered significant. We used IBM SPSS Statistics for Windows 22.0 (IBM, USA) for data analysis.

## Results

We could obtain initial diagnoses/hypotheses from completed prehospital emergency care reports in 835 emergency patients presenting with CUE (45% female, 55% male). Median age was 65 yrs., interquartile range (IQR) 47–75. Median GCS was 6 (IQR 3–8). Overall, 1217 initial diagnoses/hypotheses were stated. Three hundred-one out of 835 patients (36%) had more than one recorded initial diagnosis/hypothesis. Emergency physicians were called in for 700 patients (84%) while paramedics alone cared for 135 patients (16%).

**Fig. 2:**
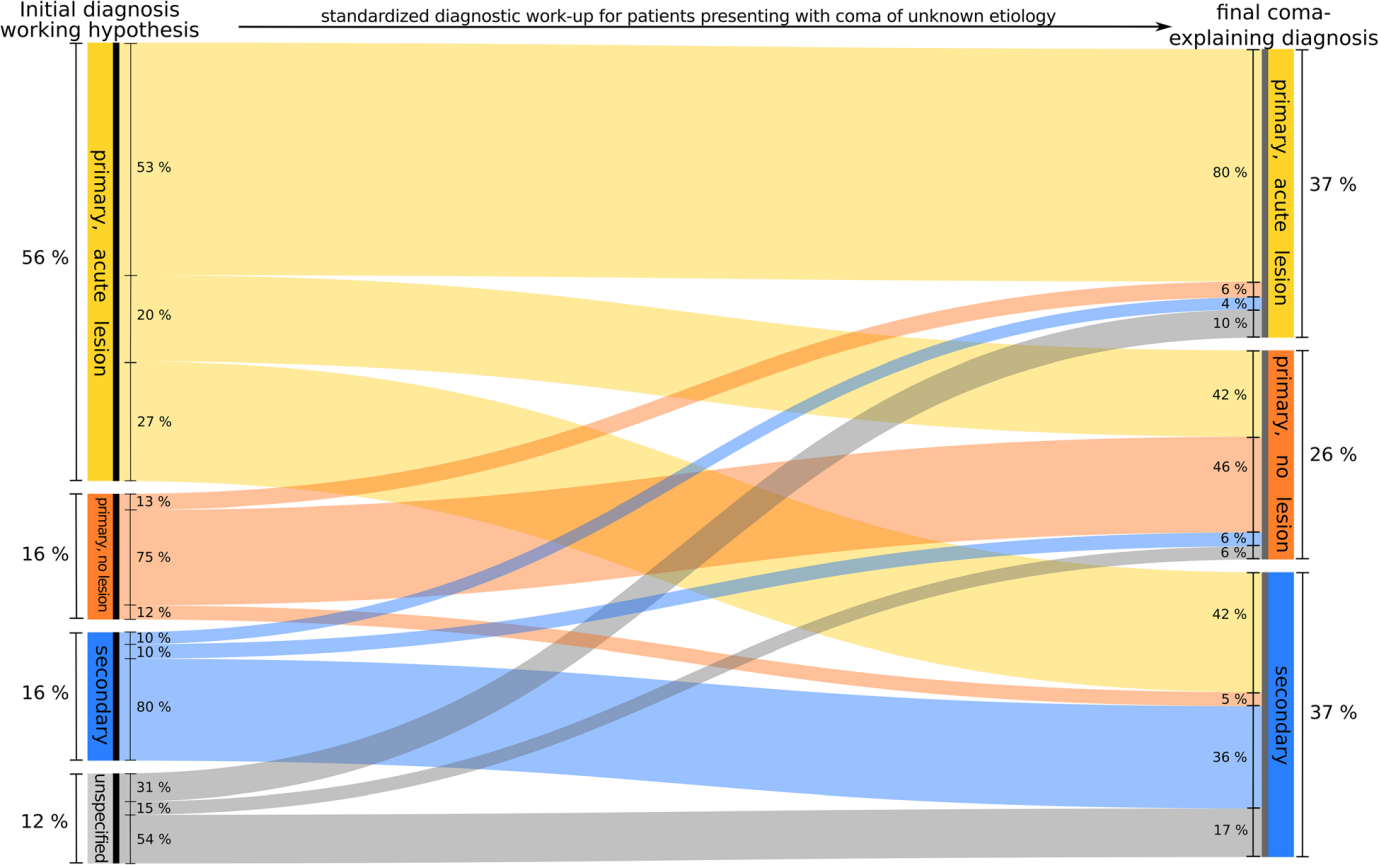
Comparison between initial diagnoses/working hypotheses and final diagnoses (grouped and classified)

Figure 2 shows the results of the comparison of categorized diagnoses/hypotheses. Four hundred sixty-eight patients (56%) were grouped into class I by their initial diagnoses/hypotheses. Slightly more than half of these patients matched the final class of diagnoses (match: 248 [53%]). However, 92 patients (20%) were class II at discharge and 128 (27%) were class III at discharge.

One hundred thirty-five patients (16.2%) had initial diagnose/hypotheses s from class II. One hundred and two of these (75%) matched the class of final diagnoses. However, 17 patients (13%) were class I at discharge and 16 (12%) were class III at discharge.

We found the highest match in initial diagnoses/hypotheses from class III (136 patients [16.3%]). Among these patients, 109 (80%) matched class III of final diagnoses. Final diagnoses of 13 patients (10%) were from class I while final diagnoses of 14 patients (10%) were from class II.

The remaining 96 patients (11.5%) were grouped into class IV (CUE without initial diagnosis/hypothesis). In these cases, final diagnoses were from class I in 30 patients (31%), from class II in 14 patients (15%) and from class III in 52 patients (54%).

Overall, class of initial diagnosis/hypothesis and class of final diagnosis matched in 460 patients (55%). Omitting class IV patients (as there is no logical equivalent in the classification of final diagnoses), the agreement was 62% (460 of 739 patients). Cohen’s Kappa showed a value of κ = .415 (95% CI .361–.469, *p* < .005).

In 135 patients who had been cared for by paramedics alone, we found an agreement of 43% (58 of 135 patients) between working hypotheses and final diagnoses. When leaving out class IV, agreement was at 66% (58 of 88 patients). Cohen’s Kappa showed a value of κ = .462 (95% CI .384–.540, *p* < .005).

In 700 patients, emergency physicians had stated an initial diagnosis. Here, agreement between initial and final diagnosis class was at 57% (402 of 700 patients). Disregarding class IV, we found an agreement of 62% (402 of 651 patients). In these cases, Cohen’s kappa was κ = .404 (95% CI .377–.431, *p* < .005).

Table 1 displays the comparison of grouped diagnoses/hypotheses in all cases. In 98 patients, emergency care providers stated more than one diagnosis/hypothesis. We found a percent agreement of 39% (332 matches in 835 patients).

**Table 1:**
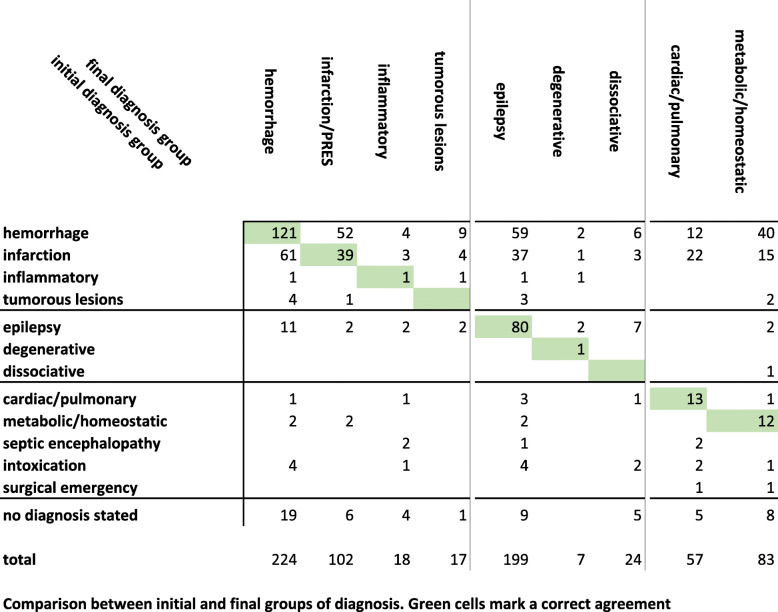
Comparison between initial diagnoses/working hypotheses and final diagnoses (grouped and classified). Green cells indicate agreement

Table 2 shows differences in the distribution of initial diagnoses/hypotheses in relation to binned GCS values. Differences were significant (χ^2^-test: *p* = .002). The extent of difference was weak (Cramér V = .111, *p* < .005).

**Table 2:** Distribution of initial diagnoses/working hypotheses in 835 patients (grouped and classified), ordered by GCS bins. Percent values in rows

GCS	number of patients				
	Class I	Class II	Class III	Class IV	Total
3–5	247 (61%)	53 (13%)	74 (18%)	34 (8%)	408
6–8	131 (54%)	49 (20%)	34 (14%)	29 (12%)	243
9–12	90 (49%)	33 (18%)	28 (15%)	33 (18%)	184

Overall, in-hospital mortality was 23.4% (*n* = 195). The subgroup of patients with initial diagnoses/hypotheses from class I showed the highest mortality. Almost one third of these patients died in hospital (31.4%). Accordingly, in-hospital mortality was 8.9% in the subgroup of initial diagnoses/hypotheses from class II, 12.5% in subgroup of initial diagnoses/hypotheses from class III and 19.8% in subgroup of initial diagnoses/hypotheses from class IV. By contrast, final diagnoses were associated with in-hospital mortality rates of 47.1% (class I), 5% (class II) and 12.8% (class III). Binary logistic regression showed higher GCS bins to be associated with lower in-hospital mortality (OR .503, 95% CI .387–.654). Older age was associated with higher in-hospital mortality (OR 1.048, 95% CI 1.034–1.061). The nominal variable sex did not significantly interact with in-hospital mortality (OR 1.135, 95% CI .773–1.668).

We investigated the association between class of initial diagnosis/hypotheses and mortality to see if the initial diagnosis/hypotheses might have identified critically ill patients disregarding the factual diagnosis. The four classes did not significantly interact with in-hospital mortality (Class I: OR .982, 95% CI .518–1.836; Class II: OR .932, 95% CI .326–2.211; Class III: 1.069, 95% CI .465–2.459, class IV was the reference variable). The class of final diagnosis was significantly associated with in-hospital mortality (class I: OR 5.425, 95% CI 3.409–8.633, class II: OR .354, 95% CI .163–.77, class III was the reference variable).

Regarding cases cared for by paramedics alone, only age (OR 1.128, 95% CI 1.048–1.214) and class I of final diagnoses (OR 12.383, 95% CI 1.772–86.525) did interact significantly with in-hospital mortality.

In the 700 cases accompanied by an emergency physician, class I of final diagnoses (OR 5.246, 95% CI 3.167–8.689), class II of final diagnoses (OR .339, 95% CI .149–.773), GCS bin (OR 0.53, 95% CI .402–.698) and age (OR 1.042, 95% CI 1.028–1.056) interacted significantly with mortality. Classes of initial diagnoses/hypotheses were not associated significantly with in-hospital mortality.

## Discussion

The primary objective of this study was to evaluate the accuracy of initial diagnoses or working hypotheses in CUE given by prehospital emergency care providers, since these often set the course of management in this high-risk group. We could not detect a sufficient degree of accuracy between initial diagnoses/hypotheses and final diagnoses in a large cohort of emergency patients presenting with CUE. We performed a sub-group analysis to detect possible differences in diagnostic accuracy between different levels of emergency care (paramedics versus emergency physicians). Here, we did not find relevant differences as is expressed by similar κ-values and confidence intervals. When omitting class IV patients (CUE without initial diagnosis/hypothesis), percent agreement between initial diagnoses/hypotheses and final diagnoses was lower in the sub-group of emergency physicians than in the sub-group of paramedics. However, the proportion of class IV patients was higher in the paramedic group. Paramedics may have tended to flag uncertain cases simply as “comatose of unknown etiology” rather than providing a working hypothesis. We believe this to be the most likely explanation for Cohen’s Kappa being somewhat lower in the sub-group of emergency physicians. This sub-group analysis points out that the inability of initial diagnoses (or working hypotheses, respectively) to correctly predict the underlying causes of CUE is much rather related to the symptom per se than to differences in training and competence of emergency care providers.

To our knowledge, this is the first study on the accuracy of initial diagnoses/hypotheses in CUE patients. However, several studies investigated this topic in more general populations of emergency patients, regardless of their presenting complaint. In a study on 596 patients, Heuer et al. showed a match of 91% between initial prehospital diagnosis class and final diagnosis class [19]. Arntz et al. also investigated initial diagnosis accuracy and found a match of 90% in 2.254 patients, using 139 simplified diagnoses [20]. Sporer et al. reported on the accuracy of initial diagnoses made in the ED in patients with a GCS ≤14 [13]. Accordance between initial and final diagnosis ranged from 25 to 78% depending on the physician’s confidence in his diagnosis. The mean accuracy was 60.2%.

In our study on CUE patients only, we found that in 39% percent of patients, initial diagnoses/hypotheses matched the group of final diagnoses (e.g., haemorrhage) while 62% of initial diagnoses/hypotheses matched the class of final diagnoses (e.g., primary CNS pathology with acute structural damage). There are several reasons to explain this less-than-desirable level of accuracy in our cohort. The main reason is the wide spectrum of different pathologies possibly causing the emergency symptom CUE. In other cardinal symptoms, reaching a higher accuracy may be much more likely because the presenting symptom may have fewer differential diagnoses (e.g., stroke). Second, the comatose patient’s inability to give a history and participate in their examination impedes a correct diagnosis. This is also seen in the mistriage of “found down patients” to an initially surgical or medical evaluation [21].

In our study, primary CNS pathologies with acute brain damage have been highly overestimated by prehospital care providers. Overestimation is not uncommon and is also seen in other emergencies [22–24]. A patient appearing severely ill seemingly encouraged formulating an initial diagnosis from class I. This may be seen in the association between low GCS values and a higher proportion of initial diagnoses/hypotheses from class I. However, GCS only quantifies the symptom and cannot detect the underlying pathology. Furthermore, around 19.5% of all patients in our study with an acute primary brain lesion were not flagged with the appropriate initial diagnosis or working hypotheses although acute primary brain lesions are less prevalent than suspected. Given the highest mortality, this mismatch puts these patients at risk. We believe that overestimation in order to indicate a high priority patient, regardless of the factual diagnosis, can be a systematic flaw. Apparently, only a severe diagnosis obtains high priority, which leads emergency care providers to suspect severity. Emergency care providers are typically aware of the uncertainty [25] and want to initiate a fast work-up in the emergency department. However, initial diagnoses/hypotheses are no appropriate tool for detecting high priority patients, as seen in failure to predict in-hospital mortality by the initial diagnosis/hypothesis.

The symptom CUE in itself must be considered a life-threatening emergency, regardless of initially suspected diagnoses. As we could show, initial diagnoses or working hypotheses are both insufficient predictors of factual underlying diagnoses and they do not reliably detect high-priority patients, regardless of different care providers’ qualifications. Thus, emergency work-up should not rely on any initial diagnosis or working hypothesis because these depend on diagnostic information that is hardly available in the early stages of management of a comatose patient. We therefore strongly advocate for a standardized diagnostic work-up for all CUE patients that should be triggered by the emergency symptom alone and not by any suspected diagnosis [26]. This work-up should take into account the wide range of possible coma-underlying pathologies. A standardized routine should always be completed – even when a certain coma-explaining pathology may be seemingly evident. Providing professional life support to these critically ill patients, pre-hospital emergency care providers should transfer these patients to specialist centres that have direct access to diagnostic and therapeutic facilities from all medical and surgical specialities required.

### Limitations

Like all retrospective analyses, we relied on the quality of data recording which can be especially difficult in an emergency. The need to categorize, generalize and hierarchize the initial diagnoses recorded by emergency care providers requires a classification. Smaller classes are closer to the single diagnoses, however we tried to describe three clinically relevant, but broader classes. The dynamics in acute emergency disorders may result in patients presenting in different clinical states at different times [27]. The direction of this bias disadvantages the match between initial and final diagnosis. In our tertiary care centre in a city with a high density of hospitals, inclusion bias may provoke a shift to more severe disorders than in other hospitals. Emergency care providers might have brought in fewer apparently obvious cases which might also have disadvantaged the match between initial and final diagnoses. Furthermore, results should ideally be reproduced in multicentric studies.

## Conclusion

The initial diagnosis in CUE is unreliable and therefore an inappropriate decision-making tool, both for guiding the way to the correct diagnosis and for detecting the especially critical patient. Although initial diagnoses are invaluable in the general clinical routine, in CUE they are false friends: They may narrow down the broad spectrum of possible etiologies too early in the diagnostic process and lead to premature conclusions. However, finding the correct diagnoses is the bottleneck before starting time-critical therapies. Thus, emergency work-up of CUE should not rely on any initial diagnosis, rather every comatose patient should undergo a fast and thorough standard work-up.

## Data Availability

The anonymized datasets analysed in this study are available from the corresponding author on reasonable request.

## Abbreviations

CNS: Central Nervous System
CUE: Coma of unknown Etiology
CSF: Cerebrospinal Fluid
ED: Emergency Department
EMS: Emergency Medical Service
GCS: Glasgow Coma Score
IQR: Interquartile Range
OR: Odds Ratio
CI: Confidence Interval
